# Supplementary material for: Teosinte Pollen Drive guides maize diversification and domestication by RNAi
Source: bioRxiv. 2023 Nov 10:2023.07.12.548689. Originally published 2023 Jul 13. Preprint. [Version 3] doi: 10.1101/2023.07.12.548689 (PMC10370002; doi:10.1101/2023.07.12.548689)
Supplement: Supplement 2 [file media-2.pdf]

Supplementary tables for

Teosinte Pollen Drive guides maize domestication and evolution by RNAi.

Authors:

Benjamin Berube[1], Evan Ernst[1], Jonathan Cahn[1], Benjamin Roche[1], Cristiane de Santis Alves[1], Jason Lynn[1], Armin Scheben[2], Adam Siepel[2], Jeffrey Ross-Ibarra[3], Jerry Kermicle[4] and Rob Martienssen[1*]

[1] Howard Hughes Medical Institute, Cold Spring Harbor Laboratory, Cold Spring Harbor NY11724
[2] Simons Center for Quantitative Biology, Cold Spring Harbor Laboratory, Cold Spring Harbor NY11724
[3] Dept. of Evolution & Ecology, Center for Population Biology and Genome Center, University of California, Davis CA
[4] Laboratory of Genetics, University of Wisconsin, Madison WI

Supplementary Table 1: Phenotypic Segregation Ratios for Reciprocal *TPD* Crosses

Cross	Fertile	Semi-Sterile	Sterile	Chi-square
TPD ♂	0	142	0	$\chi^2(2:1:1) = 164.29$, $p < 0.00001$
TPD ♂	0	179	0	$\chi^2(2:1:1) = 209.29$, $p < 0.00001$
TPD ♂	0	219	0	$\chi^2(2:1:1) = 257.27$, $p < 0.00001$
TPD ♀	144	89	58	$\chi^2(2:1:1) = 3.301$, $p = 0.192$
TPD ♀	159	109	71	$\chi^2(2:1:1) = 5.234$, $p = 0.073$
TPD ♀	106	60	54	$\chi^2(2:1:1) = 0.193$, $p = 0.908$

Supplementary Table 2: Genotypic Segregation Ratios for Reciprocal *TPD* Crosses

Parent	*tpd1; tpd2*	*Tpd2+/-*	*Tpd/++*	*Tpd1+/-*	Chi-square
TPD ♂	0	0	142	0	$\chi^2(1:1:1:1) = 162.44$, $p < 0.00001$
TPD ♂	0	0	179	0	$\chi^2(1:1:1:1) = 204.41$, $p < 0.00001$
TPD ♂	0	0	219	0	$\chi^2(1:1:1:1) = 254.37$, $p < 0.00001$
TPD ♀	81	63	89	58	$\chi^2(1:1:1:1) = 4.447$, $p = 0.217$
TPD ♀	69	90	109	71	$\chi^2(1:1:1:1) = 6.029$, $p = 0.110$
TPD ♀	51	55	60	54	$\chi^2(1:1:1:1) = 0.378$, $p = 0.945$

Supplementary Table 3: Genome Assembly Metrics. Values calculated on scaffolded assemblies with contigs split by 10 or more consecutive Ns. Repeat content determined by RepeatMasker with the combined MTEC repeat library "maizeTE02052020" distributed at https://github.com/oushujun/MTEC.

	**TPD**<br>**TPD 1.0 2021**	**W22**<br>**W22 Reference NRGENE-2.0 2018**
**Contigs**		
#	945	49,111
Longest (Kbp)	44,353	831
N50 (Kbp)	7,744	88
**Scaffolds**		
# > 10Mbp	10	10
Span (Mbp)	2,112	2,134
Longest (Mbp)	306	311
Gap bases (%)	0.02	1.90
**Annotations**		
Coding Genes (W22 liftover)	40,564	40,961
Interspersed Repeats (%)	81.37	80.04
CentC masked (Kbp)	2,876	137
**QC**		
merqury QV estimate	34.2	-
Complete BUSCOs (%)	90.6	91.1
Missing BUSCOs (%)	0.3	0.6

Supplementary Table 4: Empirical *p*-values for selection scan with a windowed weighted F_{ST} statistic for *Teosinte Pollen Drive*-linked (*TPD*-linked) regions and a validation set of domestication genes. See Supplementary Table 5 for gene coordinates. Empirical *p*-values for each gene are shown for the overlapping window with the lowest *p*-value. Significant *p*-values <0.05 are indicated with an asterisk for both unadjusted and Bonferroni-adjusted values accounting for each population pair tested.

**Group**	**Gene**	***mexicana* - Landrace**	***parviglumis* - Landrace**	**Landrace - Modern**	***mexicana* - Modern**	***parviglumis* - Modern**
		**Windowed F_{ST} empirical *p*-values**				
TPD-linked	*dcl2*	0.321	0.584	0.479	0.132	0.345
TPD-linked	hairpin	0.305	0.503	0.742	0.106	0.255
TPD-linked	*rdm1*	0.510	0.660	0.326	0.585	0.802
TPD-linked	*tdr1*	0.263	0.324	0.166	0.096	0.124
Domestication	*gt1*	0.128	0.058	0.020*	0.213	0.164
Domestication	*tb1*	0.016*	0.110	0.329	0.004*	0.047*
Domestication	*tga1*	0.032*	0.003*	0.356	0.021*	0.004*
Domestication	*zagl1*	0.025*	0.001*	0.201	0.003*	0.001*
		**Windowed F_{ST} empirical adjusted *p*-values**				
TPD-linked	*dcl2*	1.000	1.000	1.000	0.662	1.000
TPD-linked	hairpin	1.000	1.000	1.000	0.529	1.000
TPD-linked	*rdm1*	1.000	1.000	1.000	1.000	1.000
TPD-linked	*tdr1*	1.000	1.000	0.828	0.482	0.622
Domestication	*gt1*	0.638	0.290	0.100	1.000	0.822
Domestication	*tb1*	0.082	0.551	1.000	0.021*	0.236
Domestication	*tga1*	0.158	0.016*	1.000	0.105	0.020*
Domestication	*zagl1*	0.123	0.004*	1.000	0.013*	0.003*

Supplementary Table 5: Selection scan with a windowed |iHS| statistic in *Teosinte Pollen Drive*-linked (*TPD*-linked) regions and a validation set of domestication genes in teosinte and maize populations. Counts of individual significant (*p*<0.05) SNPs as well as empirical *p*-values for 10kb windows are shown. Genomic coordinates for *dcl2* (Zm00001eb219690), *tdr1* (Zm00001eb224090) and *rdm1* (Zm00001eb275620) are for the public maize B73 NAMv5 reference genome and annotation from MaizeGDB. Coordinates for the domestion genes are based on the same assembly and annotation. Significant *p*-values <0.05 are indicated with an asterisk. SNP counts and per window *p*-values are shown as unadjusted and Bonferroni-adjusted values accounting for each population tested. Cases in which there was insufficient data to calculate a *p*-value are shown as "NA".

Group	Gene	Genomic coordinates	***mexicana***	***parviglumis***	**Landrace**	**Modern**
			**Count of |iHS| significant SNPs**			
TPD-linked	*dcl2*	Chr5:20,831,070-20,848,118 (-)	46	6	1	18
TPD-linked	*tdr1*	Chr5:40,759,118-40,761,756 (-)	1	0	0	0
TPD-linked	hairpin	Chr5:95,990,889-96,002,706 (+)	4	30	1	0
TPD-linked	*rdm1*	Chr6:107,817,218-107,820,394 (+)	0	2	0	14
Domestication	*zagl1*	Chr5:20,831,070-20,848,118 (-)	48	40	0	0
Domestication	*gt1*	Chr5:40,759,118-40,761,756 (-)	0	0	0	3
Domestication	*tb1*	Chr5:95,990,889-96,002,706 (+)	5	2	2	0
Domestication	*tga1*	chr1:272330564-272332648 (+)	2	0	0	0
			**Count of |iHS| adjusted significant SNPs**			
TPD-linked	*dcl2*	Chr5:20,831,070-20,848,118 (-)	10	0	0	0
TPD-linked	*tdr1*	Chr5:40,759,118-40,761,756 (-)	0	0	0	0
TPD-linked	hairpin	Chr5:95,990,889-96,002,706 (+)	0	10	0	0
TPD-linked	*rdm1*	Chr6:107,817,218-107,820,394 (+)	0	0	0	6
Domestication	*zagl1*	Chr1:4932248-4948340 (-)	0	12	0	0
Domestication	*gt1*	chr1:23433554-23435122 (+)	0	0	0	3
Domestication	*tb1*	chr1:272330564-272332648 (+)	4	1	2	0
Domestication	*tga1*	chr4:46647932-46652896 (+)	0	0	0	0
			**Windowed |iHS| empirical *p*-values**			
TPD-linked	*dcl2*	Chr5:20,831,070-20,848,118 (-)	0.008*	0.341	0.316	0.079

TPD-linked	*tdr1*	Chr5:40,759,118-40,761,756 (-)	0.377	0.649	0.759	0.468
TPD-linked	hairpin	Chr5:95,990,889-96,002,706 (+)	0.216	0.171	0.493	0.215
TPD-linked	*rdm1*	Chr6:107,817,218-107,820,394 (+)	0.421	0.414	0.737	0.021*
Domestication	*zagl1*	Chr5:20,831,070-20,848,118 (-)	0.020*	0.039*	NA	NA
Domestication	*gt1*	Chr5:40,759,118-40,761,756 (-)	0.913	0.373	0.780	0.410
Domestication	*tb1*	Chr5:95,990,889-96,002,706 (+)	0.021*	0.116	0.054	0.188
Domestication	*tga1*	chr1:272330564-272332648 (+)	0.315	0.477	0.641	0.673
			**Windowed |iHS| empirical adjusted *p*-values**			
TPD-linked	*dcl2*	Chr5:20,831,070-20,848,118 (-)	0.031*	1.000	1.000	0.314
TPD-linked	*tdr1*	Chr5:40,759,118-40,761,756 (-)	1.000	1.000	1.000	1.000
TPD-linked	hairpin	Chr5:95,990,889-96,002,706 (+)	0.863	0.686	1.000	0.861
TPD-linked	*rdm1*	Chr6:107,817,218-107,820,394 (+)	1.000	1.000	1.000	0.085
Domestication	*zagl1*	Chr1:4932248-4948340 (-)	0.080	0.156	NA	NA
Domestication	*gt1*	chr1:23433554-23435122 (+)	1.000	1.000	1.000	1.000
Domestication	*tb1*	chr1:272330564-272332648 (+)	0.082	0.465	0.217	0.753
Domestication	*tga1*	chr4:46647932-46652896 (+)	1.000	1.000	1.000	1.000

Supplementary Table 6: Genotyping markers used in this study. The "type" column refers to the general marker design associated with the primer sequence. RFLP markers will denote the associated restriction enzyme in the "type" column.

Name	Type	Sequence (5'-3')	WT	Mut/*TPD*
dcl2^{T}f	BstNI	AGCGCCATTTACAATTTCAGCA	183, 211	394
dcl2^{T}-r	BstNI	TGTTGCCAGTGAATCAGCACTA	183, 211	394
m5.0-f	SSLP	TTAGTAGTGTCCTGGCGCTC	295	165
m5.0-r	SSLP	GTGAGGGACTAGGGCATGTG	295	165
m5.1-f	SSLP	CCTGCATAGAGATGCCATCAA	310	180
m5.1-r	SSLP	CGACGACGACTCATCCACGA	310	180
m5.2-f	SSLP	TGTCTTCCTCCAACTGTGCT	312	211
m5.2-r	SSLP	ACTGCCCAAAGAGCATGTGT	312	211
m5.3-f	MfeI	AATGGTGTTTTCCTTGGCATTCA	253, 249	502
m5.3-r	MfeI	GTACCATGCACTCATCCCGAA	253, 249	502
m5.4-f	TseI	GGATCATGGAGTGCGTGCAG	450	161, 289
m5.4-r	TseI	AACCAGCGCTCCTCAAAGTT	450	161, 289
m6.0-f	SSLP	ACTGAGTAACCAATGCCAGA	445	363
m6.0-r	SSLP	GCAGCCTTCCAGTTCCTGTA	445	363
m6.1-f	SSLP	GATCTACTTGCACGAGAGCACC	495	374
m6.1-r	SSLP	CGGAGTAATTTCCTTGGGACA	495	374
m6.2-f	AflIII	CTACCAACTGCTCCTGAGATGG	116, 96	212
m6.2-r	AflIII	CGTTGACGAATATTGATTGTAGCCA	116, 96	212
m6.3-f	NdeI	TTGCTCCAACCTTGTCACCT	124, 100	224
m6.3-r	NdeI	GCTATCCGCAAACAGCGAGA	124, 100	224
m6.4-f	BccI	TCCTTCTCCTCTTCCCTCGG	250	110, 140
m6.4-r	BccI	AGGAACCCTGTTTGACGATCT	250	110, 140
m6.5-f	BglIII	TCCACAGAAGGACAGCAAAAGGA	174, 65	239
m6.5-r	BglIII	TAGGGTTTGTTGTGGCTGCT	174, 65	239
m6.6-f	PvuII	GGCCAAGTTGTTCAAGAAGCAT	165, 85	254
m6.6-r	PvuII	GCGTGGCCCCTTCTCTTATT	165, 85	254
m6.7-f	NdeI	GGGCATCGTGTCATTGAAGG	231	115, 116
m6.7-r	NdeI	TGCAACCTCTCAGGTCTAAG	231	115, 116
lbl-rgd1-f	MwoI	GCCCATCTGGATCTGAAGTC	262, 227	489
lbl-rgd1-r	MwoI	TTGGTGGCCACACTATCTCA	262, 227	489
dcl2mu1-f	Mu insertion	GTGTCCGCGTTCCAGAAGTC	906	800
dcl2mu1-r	Mu insertion	TAAAGGTTGTCCATTGGGCGTT	906	800
TIR4	TIR	GCCTCCATTTCGTCGAATCCC	-	-
TIR6	TIR	GCCTCTATTTCGTCGAATCCG	-	-
Gdsl-f	HiII check	GAGCGTCTCCTTCAACCCAA	983	-
Gdsl-r	HiII check	TCCTACTCCTCAGTTGGGGG	983	-
Dcl2-f	HiII check	GGCCTAGAATTCAGTTGCGG	717	-
Dcl2-r	HiII check	GAACACGCTTGTGTTCCTCG	717	-

Supplementary Table 7: RT-qPCR primers used in this study. All RT-qPCR primers used in this study were designed to bridge exon junctions (if present) in order to increase specificity. Expected amplicon sizes corresponding to a cDNA or gDNA template are listed.

Name	Target	Sequence	cDNA	gDNA
Dcl2q-1f	*Dcl2* – exon 1,2	CCCAAAAGGACACACAGCTTTC	79	159
Dcl2q-1r	*Dcl2* – exon 1,2	GCATATCTGATCTTGGAGTATGGC	79	159
Dcl2q-2f	*Dcl2* – exon 5,6	TCGACAAAAACAATGCATCTCAAAT	104	658
Dcl2q-2r	*Dcl2* – exon 5,6	GCTGAGGATCTGCAAGATGG	104	658
Dcl2q-3f	*Dcl2* – exon 18,19	CATTCTGAAGGGTGTCGGGT	174	417
Dcl2q-3r	*Dcl2* – exon 18,19	GGTCCATCACATGGTTGGGAA	174	417
Gdslq-1f	*Gdsl* – exon 1,2	GAGCGTCTCCTTCAACCCAA	81	249
Gdslq-1r	*Gdsl* – exon 1,2	ACAAGGCTACTGGCAGGTTC	81	249
Gdslq-2f	*Gdsl* – exon 2,3	CCCACCTTCGCCTTGTACTC	153	250
Gdslq-2r	*Gdsl* – exon 2,3	CCTGAACAAGAGCCTCACCC	153	250
Elfa9q-1f	*Efla9* – exon 1,2	CAAGCTGACTGTGCTGTTCTTA	181	928
Elfa9q-1r	*Efla9* – exon 1,2	GCCTTTGAATACTTGGGTGTAGTAG	181	928
Elfa9q-2f	*Efla9* – exon 1,2	CCAAGCTGACTGTGCTGTTCTT	198	945
Elfa9q-2r	*Efla9* – exon 1,2	AATCTCATCATAACGGGCCTTTGAA	198	945
Rdr6q-1f	*Rdr6* – no exons	TGTTTTGCTTCAGCTGGGGA	128	128
Rdr6q-1r	*Rdr6* – no exons	CACGCGAATGAAGCATCTGG	128	128
Rdr6q-2f	*Rdr6* – no exons	GCCTCTCACCTTTCTTGGGG	74	74
Rdr6q-2r	*Rdr6* – no exons	CCTTGAAGCTGTTGATGTGCC	74	74
Rgd1q-1f	*Rgd1* – exon 5,6	GCCACATCCTTGACTTGGCA	119	319
Rgd1q-1r	*Rgd1* – exon 5,6	TGCAGGAAGAGCGCTCAAAG	119	319
Rgd1q-2f	*Rgd1* – exon 4,6	CGCTCTTCCTGCAACAACTT	130	378
Rgd1q-2r	*Rgd1* – exon 4,6	GAGAAGCACATGGAGTACGAGG	130	378
Ago1e-1f	*Ago1e* – exon 19,20	TTCTACCTGTGCAGCCATGC	103	172
Ago1e-1r	*Ago1e* – exon 19,20	GAGTTTGCAACCCATCAGCC	103	172
Ago1e-1f	*Ago1e* – exon 10,11	GCCAAAAATTGGCCAGTGGA	128	515
Ago1e-1r	*Ago1e* – exon 10,11	TCATGACAGAAACTCCCGGC	128	515
