# Supplementary material for: Teosinte Pollen Drive guides maize domestication and evolution by RNAi
Source: bioRxiv. 2023 Aug 3:2023.07.12.548689. Originally published 2023 Jul 13. Preprint. [Version 2] doi: 10.1101/2023.07.12.548689 (PMC10370002; doi:10.1101/2023.07.12.548689)
Supplement: Supplementary file 1 [file media-1.pdf]

Extended Data for

Teosinte Pollen Drive guides maize domestication and evolution by RNAi.

Authors:

Benjamin Berube[1], Evan Ernst[1], Jonathan Cahn[1], Benjamin Roche[1], Cristiane de Santis Alves[1], Jason Lynn[1], Armin Scheben[2], Adam Siepel[2], Jeffrey Ross-Ibarra[3], Jerry Kermicle[4] and Rob Martienssen[1*]

[1] Howard Hughes Medical Institute, Cold Spring Harbor Laboratory, Cold Spring Harbor NY11724
[2] Simons Center for Quantitative Biology, Cold Spring Harbor Laboratory, Cold Spring Harbor NY11724
[3] Dept. of Evolution & Ecology, Center for Population Biology and Genome Center, University of California, Davis CA
[4] Laboratory of Genetics, University of Wisconsin, Madison WI

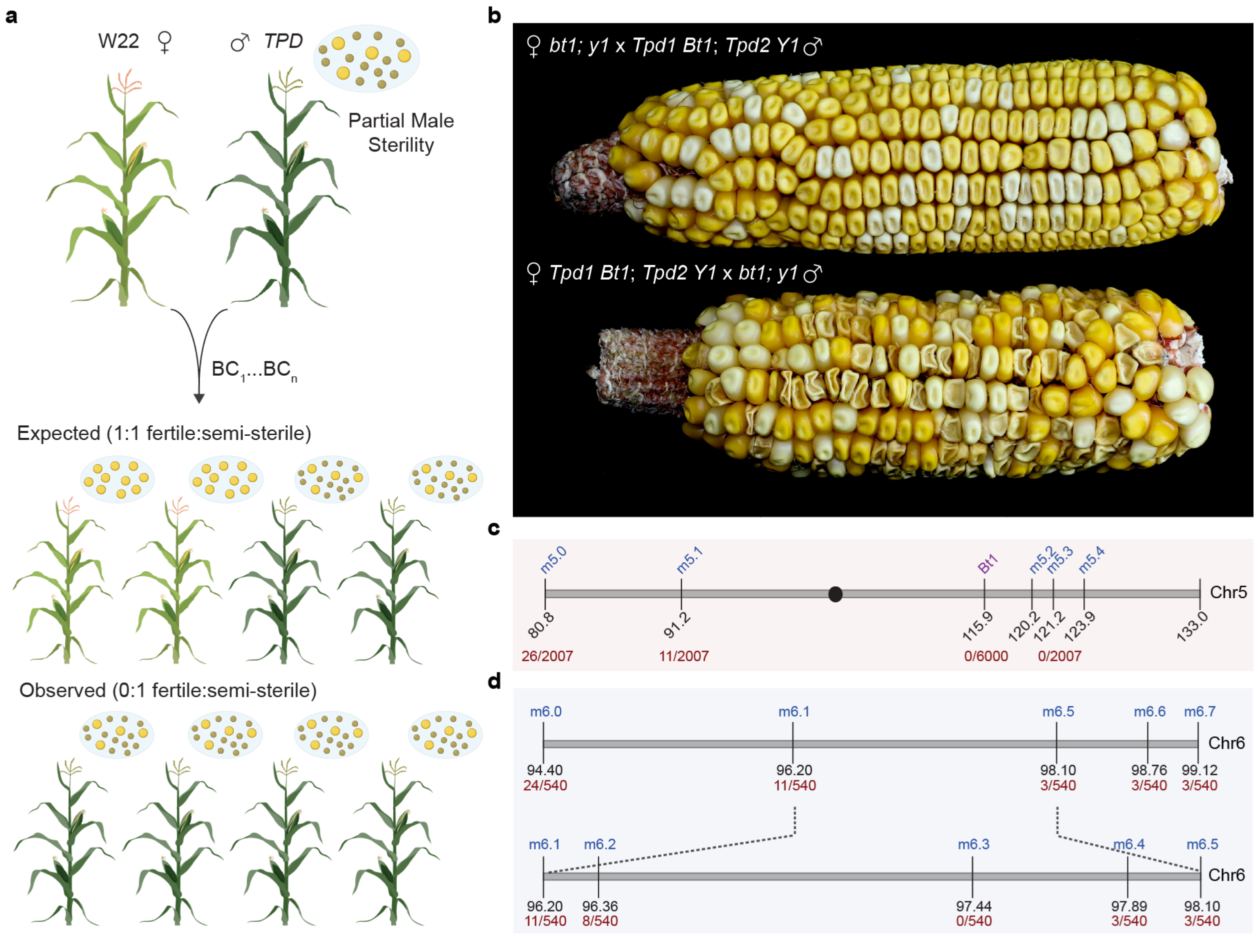

Extended Data Fig.1: *Teosinte Pollen Drive* and genetic mapping of *Tpd1* and *Tpd2*. a, Crossing scheme of the *TPD* phenotype. When back-crossed as male, all the progeny of semi-sterile *TPD* plants display the semi-sterile pollen phenotype instead of the expected 1:1 fertile:semi-sterile ratio. **b,** Representative ears from *Tpd1 Bt1/bt1*; *Tpd2 Y1/y1* reciprocal crosses with *bt1; y1* testers, demonstrating severe segregation distortion ("drive" of *Bt1*), but only through the male. *bt1 (brittle1,* collapsed kernels); *y1* (*yellow1*, white kernels). **c,** Summary of molecular and morphological mapping of the *Tpd1* interval. Molecular mapping was performed using *Tpd/++* x W22 segregating progeny, whereas morphological mapping was performed by crossing *Tpd1 Bt1/tpd1 bt1* plants to *bt1* testers. **d,** Molecular mapping of the *Tpd2* interval. SNP markers are shown in blue with recombination frequencies in red.

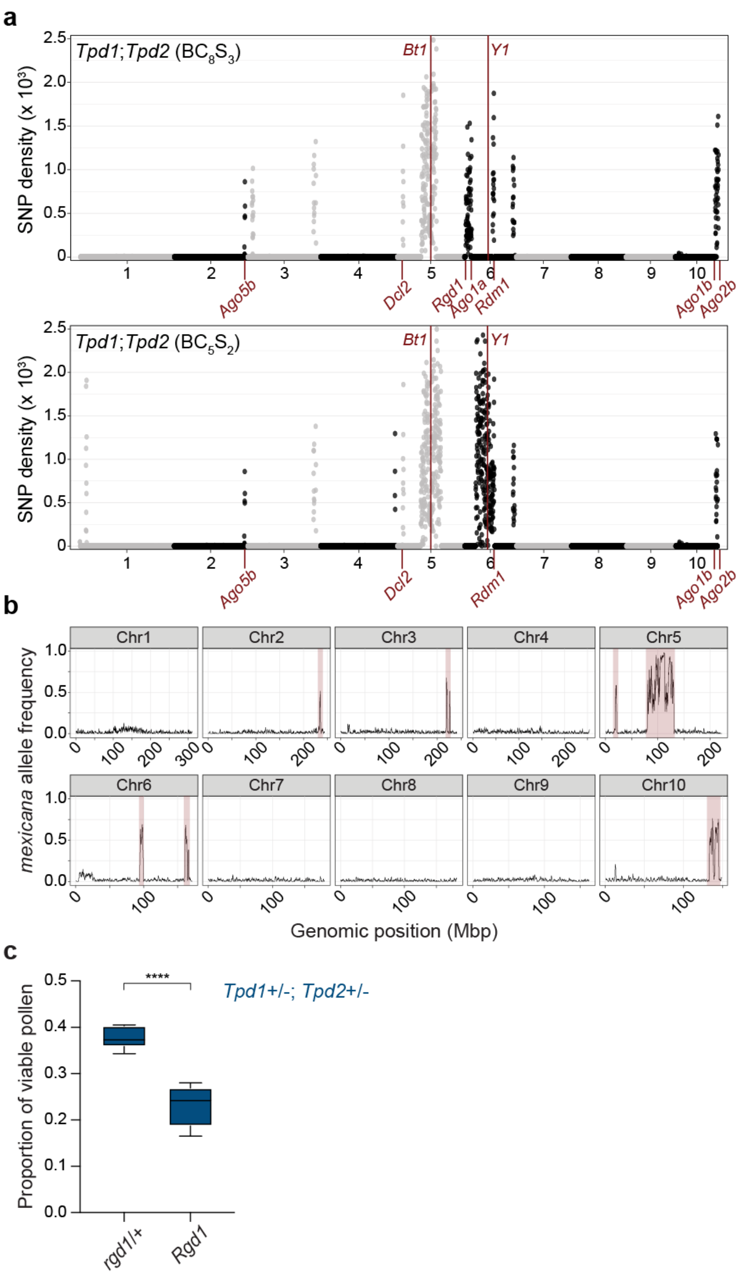

Extended Data Fig. 2: *mexicana* intervals introgressed into maize carry RNAi genes. a, Whole genome plots of homozygous *mexicana* SNP density present within *Tpd1; Tpd2* lines. The upper plot corresponds to data from bulked seedlings after 8 backcrosses and 3 self-pollinations (BC_8S_3) whereas the lower plot is from BC_5S_2 plants. SNP density is consolidated in 250Kb genomic bins. Physical locations for morphological markers *Bt1* and *Y1*, as well as *mexicana* derived RNAi genes, are labeled in red. 7/13 introgression intervals overlap in both independently maintained homozygous lines. **b,** Allele frequency at *mexicana* markers in 96 pollen grains from four different *TPD* plants subjected to single pollen grain sequencing. Regions highlighted in red were over-represented in viable pollen grains. **c,** Quantification of pollen viability in *Tpd1+/-; Tpd2+/-; rgd1/+* and *Tpd1+/-; Tpd2+/-; Rgd1* pollen demonstrating gametophytic suppression via germline segregation of the *rgd1* null allele. n ≥ 9 plants per genotype, ≥ 200 pollen grains per plant. **** $p < 0.0001$ (Welch's t-test).

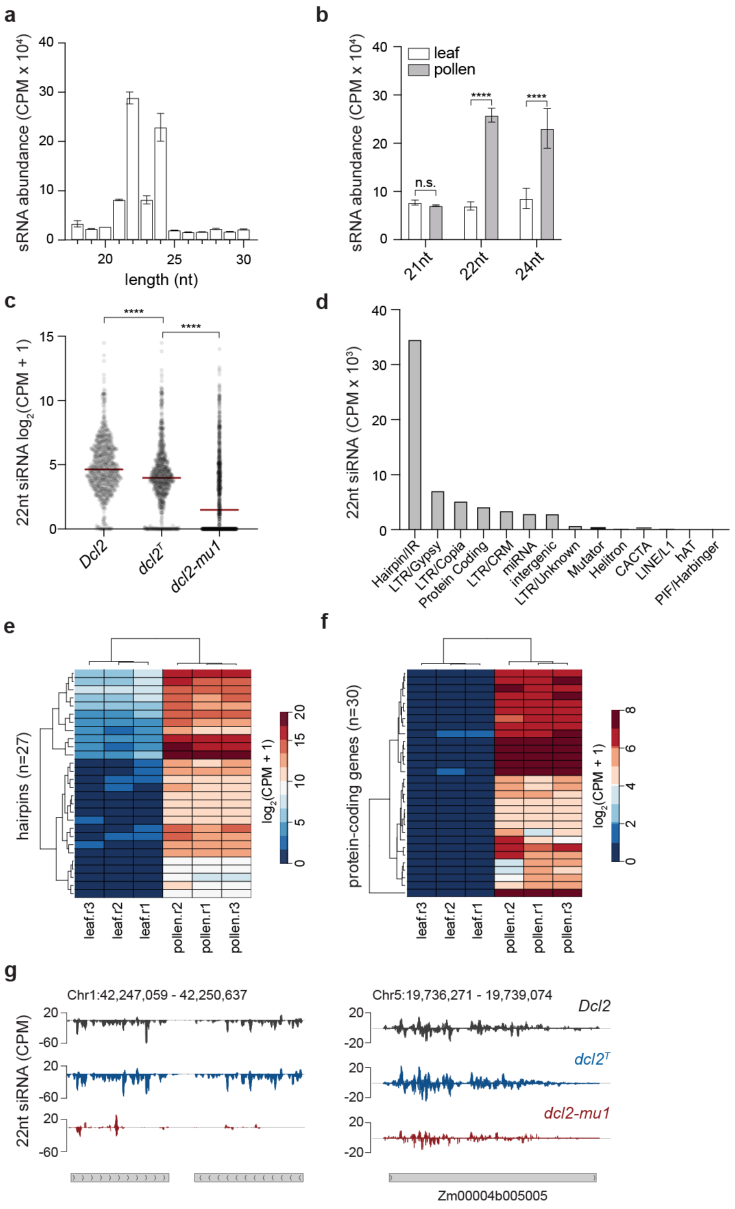

Extended Data Fig. 3: *DCL2*-dependent 22nt siRNAs from hairpins are prevalent in maize pollen. a, Distribution and relative abundances of small RNA size classes in WT pollen libraries. Bars indicate mean ± SD. n = 3 biological replicates. **b,** Comparison of relative abundances for 21nt, 22nt, and 24nt sRNA size classes in WT leaf and pollen samples. Both 22nt and 24nt sRNAs show significant increases in pollen. Bars indicate mean ± SD. n = 3 biological replicates. **** p < 0.0001 (Welch's t-test). **c,** Comparison of 22nt sRNA levels in *Dcl2*, *dcl2*^{T}, and *dcl2-mu1* pollen at 804 pollen-specific loci. Values shown are $\log_2$ transformed counts per million (CPM) averaged across replicates. n = 3 replicates per genotype. **** p < 0.0001 (ANOVA test). **d,** Summary of relative contributions for 22nt sRNA producing loci in WT pollen. Hairpin/inverted repeat (IR) hp-siRNAs represent the largest fraction of 22nt species. **e,** Heatmap showing 22nt hp-siRNA levels at hpRNA loci in leaf and pollen. **f,** Heatmap showing 22nt siRNA levels at protein-coding genes in leaf and pollen. **g,** Browser shots showing 22nt hp-siRNA accumulation at a representative hpRNA locus on chromosome 1 (left) and 22nt siRNA silencing at a representative protein-coding gene. Scale is CPM.

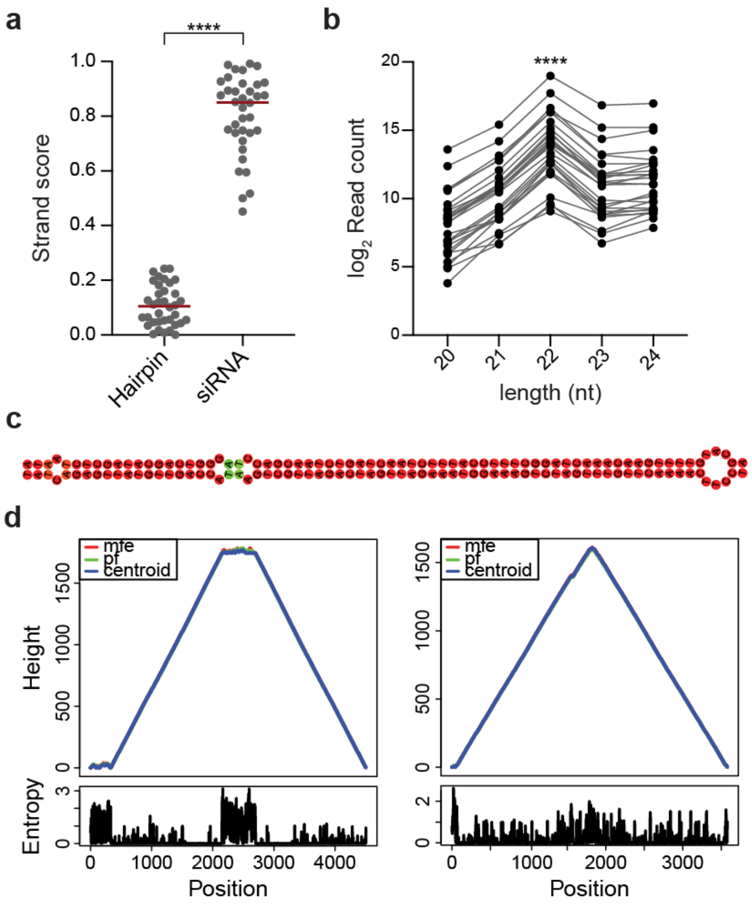

Extended Data Fig. 4: Validation of highly abundant pollen hairpin precursors. a, hp-siRNAs are expected to show strand bias. Measurement of strand score (min[plus, minus]/max[plus, minus]) at 27 putative hairpin precursors and randomly selected siRNA clusters from wild-type (W22) maize. A value of zero indicates complete strand bias, whereas a value of 1 indicates unbiased accumulation from both strands. n = 27. **** $p < 0.0001$ (Welch's t-test). **b,** log2 read count at hairpin precursors indicate 22nt size bias. n = 27. **** $p < 0.0001$ (ANOVA). **c,** Example of a 73nt stretch from a 4,480bp hairpin precursor demonstrating near-complete reverse complementarity. **d,** Mountain plots measuring thermodynamic stability of the *Tpd1* hairpin from *mexicana* and another randomly selected hairpin structure.

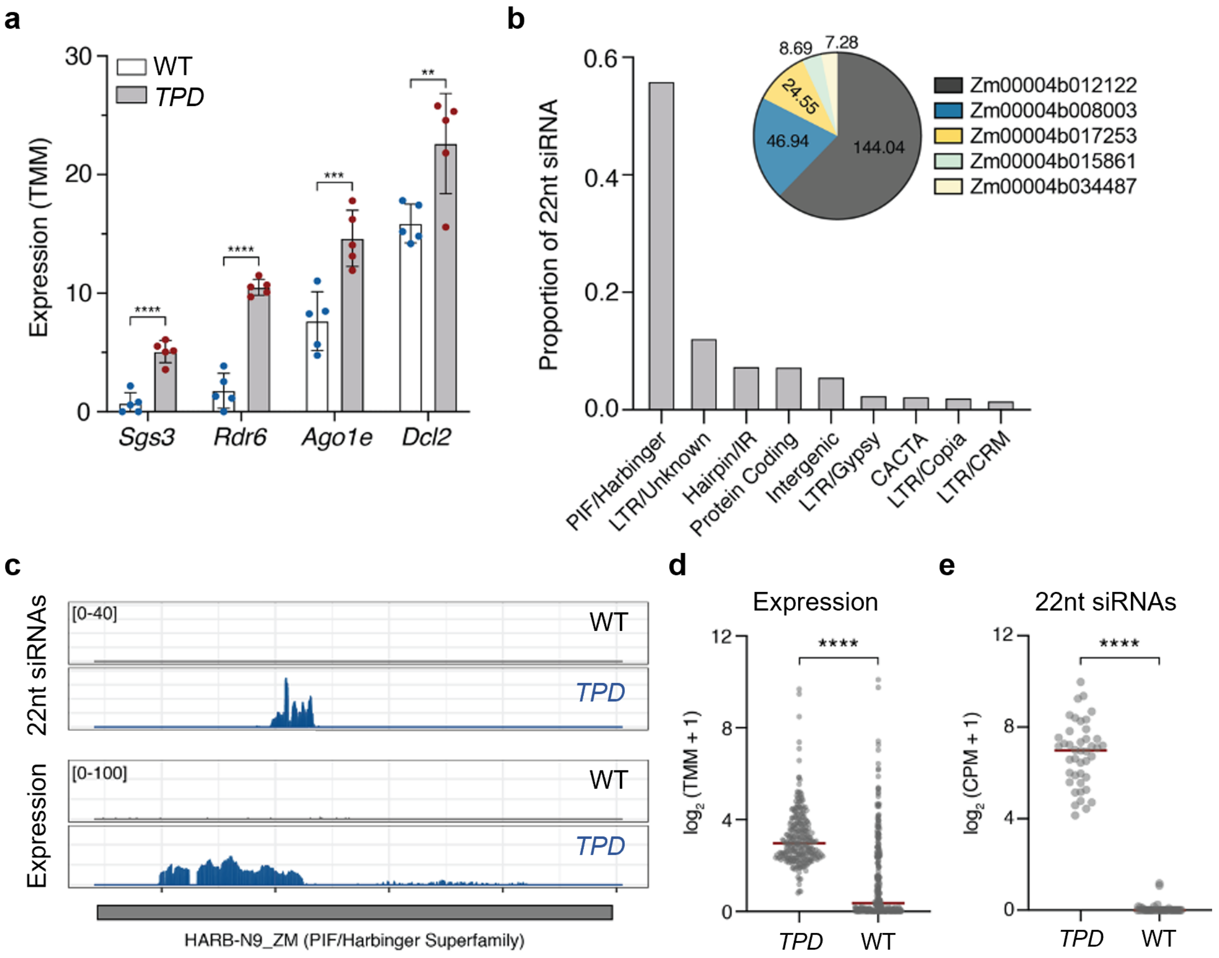

Extended Data Fig. 5: Origins and targets of 22nt small RNAs in *TPD* Pollen. a, RNAi genes associated with 22nt biogenesis and function are upregulated in *TPD* pollen. Expression is shown in TMM normalized counts. Bars show mean ± SD. n = 5 replicates per condition. **** $p < 0.0001$, *** $p < 0.001$, ** $p < 0.01$ (FDR). **b,** Relative abundances of *TPD*-dependent 22nt siRNAs mapping to annotated elements. Pie chart inset shows proportions of 22nt siRNAs targeting genes in CPM. **c,** Browser shot showing transcriptional activation at PIF/Harbinger elements in *TPD* pollen as well as 22nt siRNA accumulation. **d,** Quantification of mRNA expression at 258 PIF/Harbinger superfamily elements in WT and *TPD* pollen. **** $p < 0.0001$ (Mann-Whitney test). **e,** 22nt siRNA levels at 42 PIF/Harbinger elements in WT and *TPD* pollen. **** $p < 0.0001$ (Mann-Whitney test).

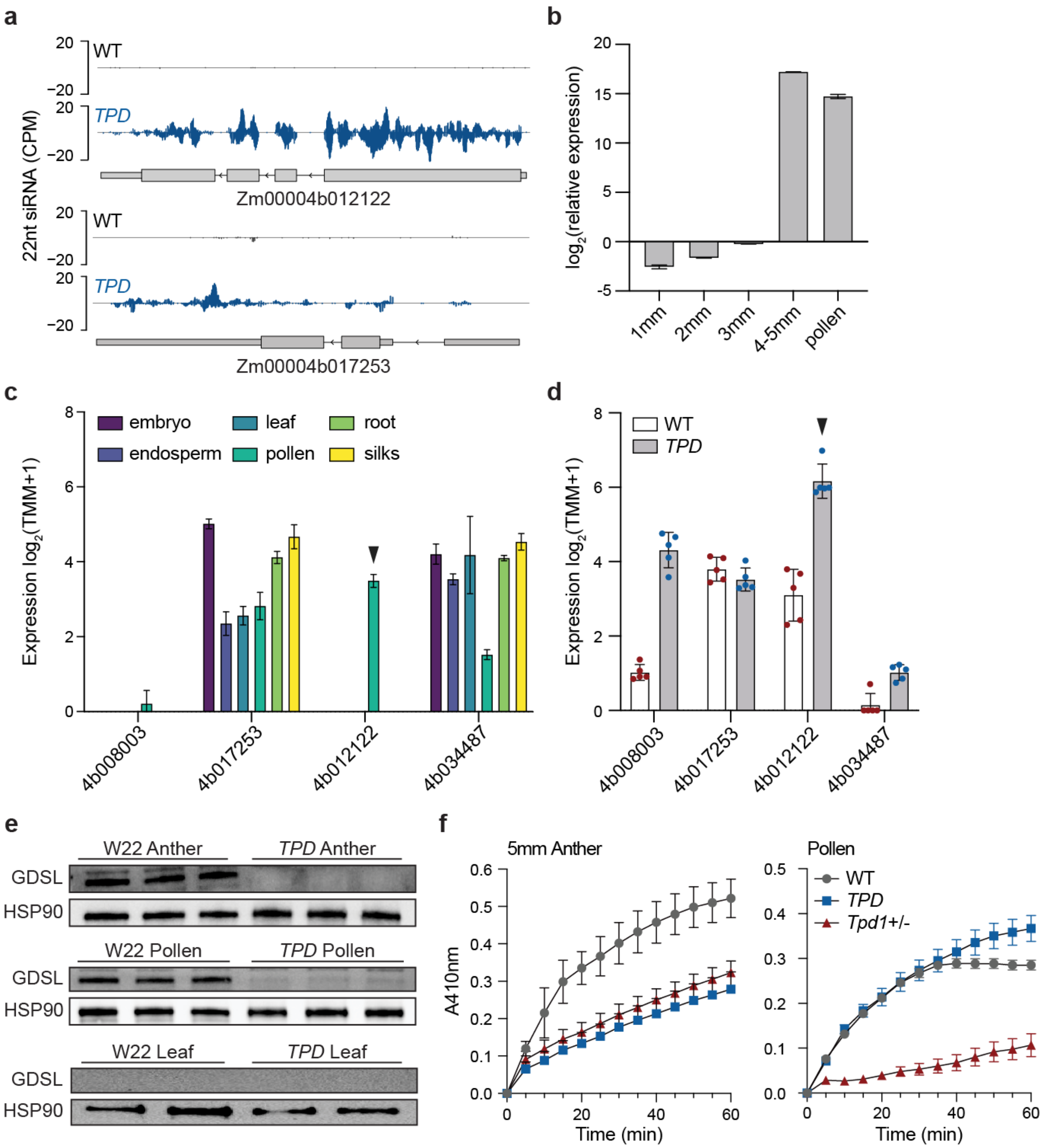

Extended Data Fig. 6: *TPD*-dependent silencing of a GDSL lipase disrupts lipid metabolism. a, Browser shots showing ectopic accumulation of 22nt siRNA at protein-coding genes in *TPD* pollen. Scale in counts per million (CPM). **b,** RT-qPCR of the 4b012122 (*Tdr1*) transcript in anther development and mature pollen. Bars show mean ± SD. n = 2 replicates per condition. **c,** RNA-seq tissue expression of 22nt siRNA targets specific to *TPD* pollen. Bars show mean ± SD. n = 3 replicates per tissue. **d,** RNA-seq expression of 22nt siRNA targets in WT and *TPD* pollen. Bars show mean ± SD. n = 5 replicates per condition. **e,** Western blot comparing TDR1 protein levels in WT and *TPD* pollen, anthers, and leaf. Protein levels were normalized using Heat Shock Protein 90 (HSP90). **f,** p-nitrophenyl butyrate esterase activity assay in 5mm anthers and pollen from WT, *TPD*, and *Tpd1*+/- plants.

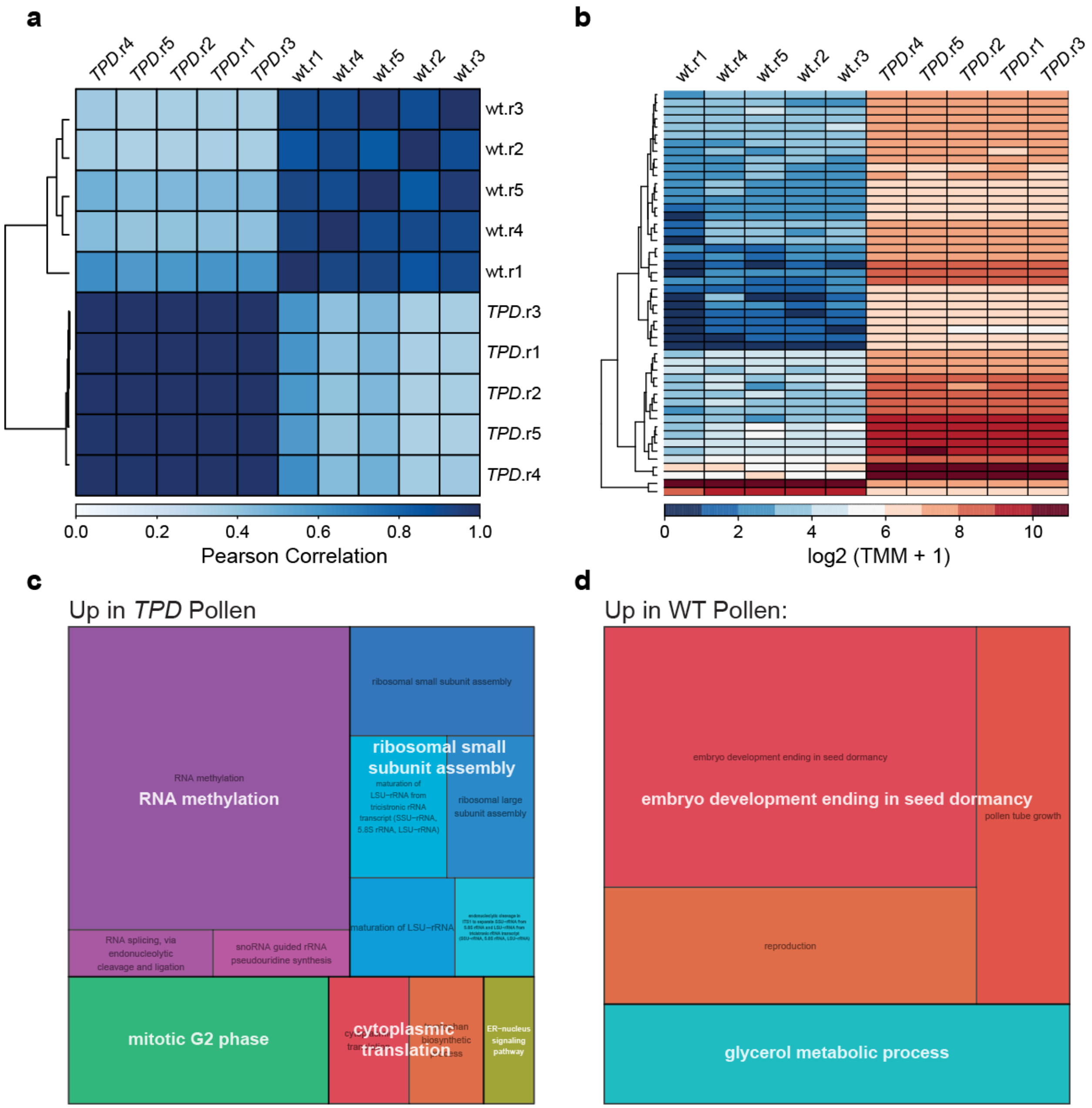

Extended Data Fig. 7: A transcriptional signature consistent with translational suppression of lipid metabolism in *TPD* Pollen. a, Hierarchical clustering based on pairwise Pearson correlations identified 1,338 upregulated genes in *TPD* pollen (log2 FC ≥ 3, FDR ≥ 0.001). **b,** Expression of the top 50 DEGs between WT and *TPD* pollen demonstrating a general up-regulation in *TPD* pollen. GO term biological processes up-regulated in **c,** *TPD* and **d,** WT pollen (FDR ≤ 0.001). Upregulated genes in *TPD* pollen were associated with RNA metabolism, ribosome assembly, and cytoplasmic translation as well as G2 mitotic arrest. This could reflect translational repression via 22nt siRNAs. Interestingly, a subset of genes associated with endoplasmic reticulum (ER)-nucleus signaling was also up-regulated (Liu and Li 2019), while genes associated with glycerol metabolism, the primary backbone for TAG synthesis, were downregulated. In pollen, the accumulation of TAGs in lipid droplets (LDs) is critical for proper membrane expansion and pollen tube growth (Taurino et al. 2018).

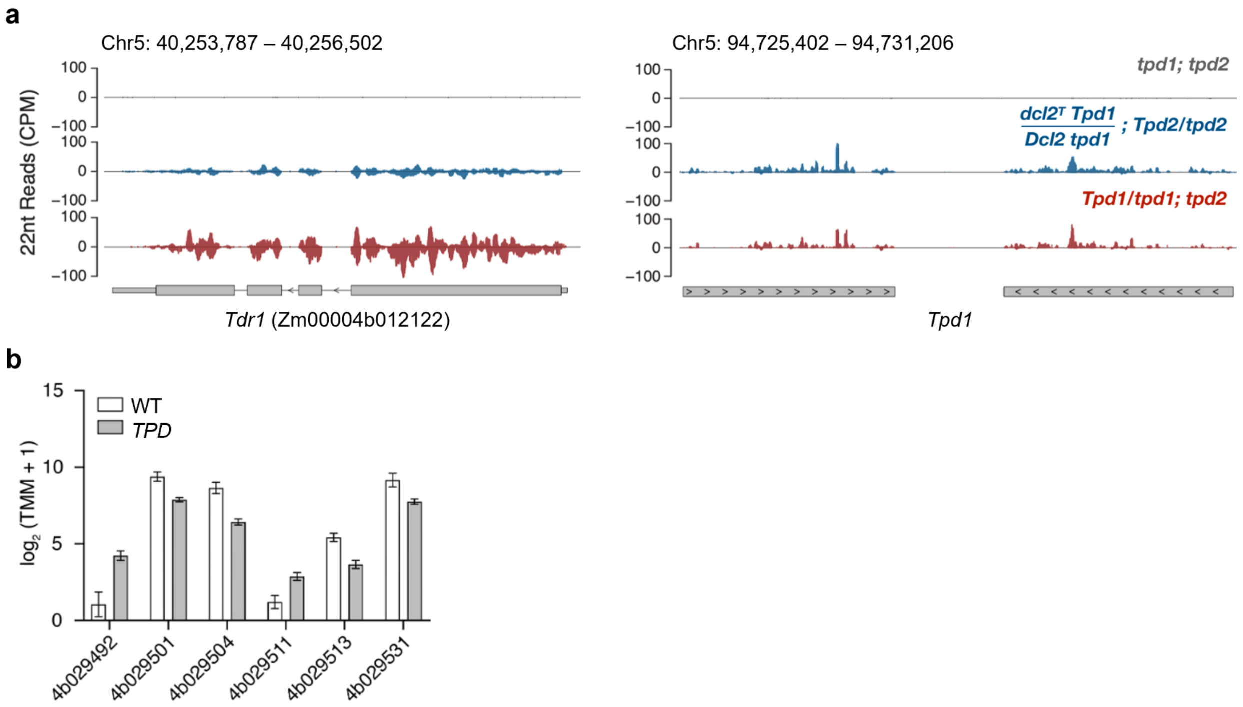

Extended Data Fig 8: *Tpd2* suppresses 22nt secondary small RNAs. a. Browser shots showing ectopic accumulation of 22nt siRNAs at *Tdr1* (left) and *Tpd1* hairpin (right) in fertile (*tpd1; tpd2*, grey), drive (*Dcl2^{T} Tpd1/Dcl2 Tpd1; Tpd2/tpd2,* blue) and sterile (*Dcl2 Tpd1/Dcl2 tpd1; tpd2*, red) pollen from maternal segregants. Scale in counts per million (CPM). *Tpd2* and *Dcl2^{T}* reduce small RNAs from *Tdr1* (left) but not from the *Tpd1* hairpin (right), consistent with a cell autonomous role in secondary small RNA biogenesis and silencing. **b.** *Rdm1* (Zm00004b029511) is one of six genes in the *Tpd2* interval expressed in pollen, and is overexpressed in *TPD* pollen.

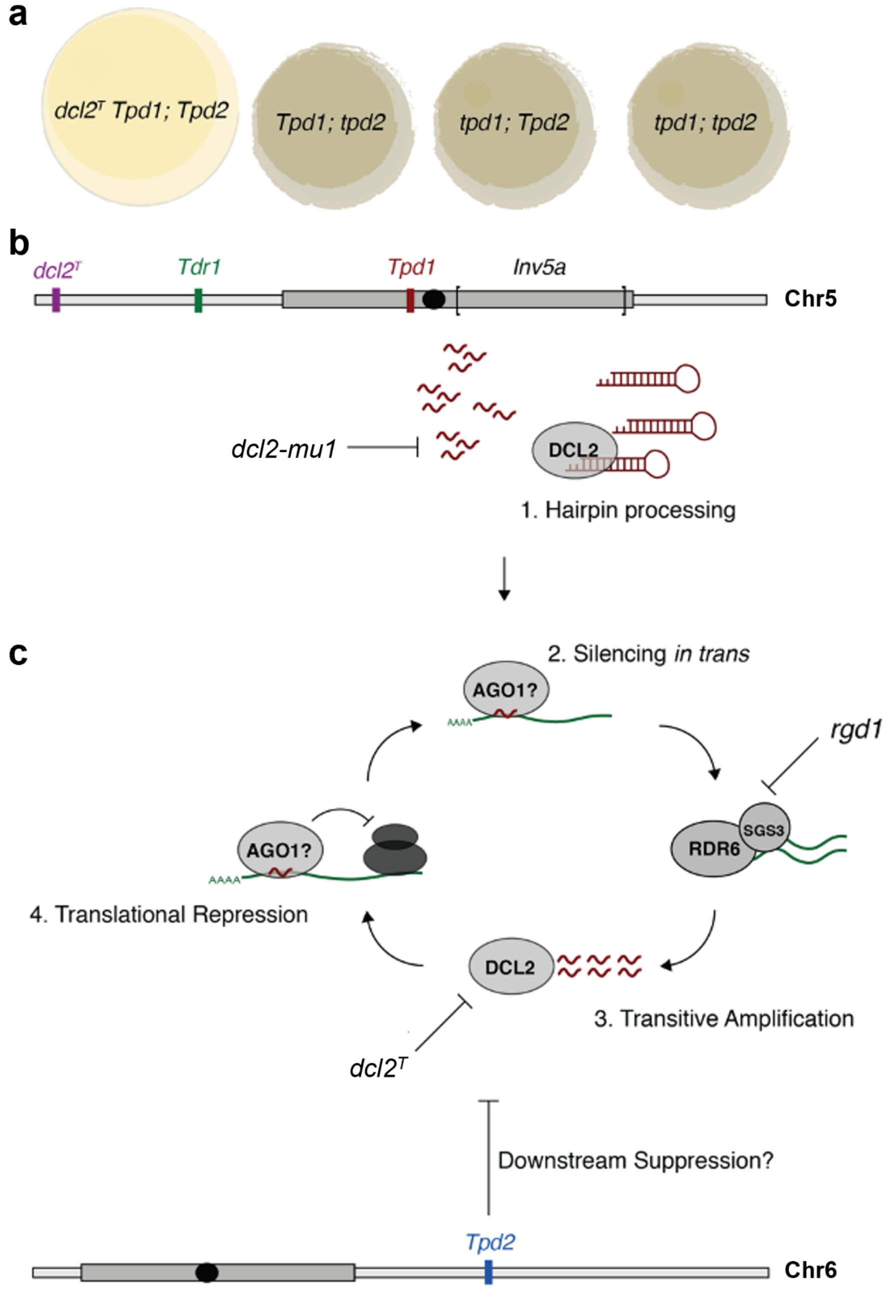

Extended Data Fig. 9: *Teosinte Pollen Drive* is mediated by RNA interference. a. The *TPD* system is defined by *mexicana* introgression intervals on chromosomes 5 and 6. Only pollen grains of the genotype *dcl2*^{T} *Tpd1*; *Tpd2* are viable, and all other competing gametes are eliminated. **b.** *Tpd1* encodes a *mexicana*-specific hairpin that produces abundant 22nt hp-siRNAs. These hp-siRNAs silence the *Tdr1* gene *in trans*, resulting in developmental arrest of pollen. *dcl2-mu1* blocks hairpin sRNA processing, restoring fertility **c.** In surviving pollen grains (yellow in **a**), genetic linkage of the *dcl2*^{T} allele limits the biogenesis of secondary 22nt sRNAs disrupting amplification of silencing, in collaboration with *Tpd2* which acts as a downstream suppressor. *rgd1* blocks secondary siRNA production and acts as an antidote.

Extended Data Table 1: Phenotypic Segregation Ratios for Reciprocal *TPD* Crosses

Cross	Fertile	Semi-Sterile	Sterile	Chi-square
TPD ♂	0	142	0	$\chi^2(2:1:1) = 164.29$, $p < 0.00001$
TPD ♂	0	179	0	$\chi^2(2:1:1) = 209.29$, $p < 0.00001$
TPD ♂	0	219	0	$\chi^2(2:1:1) = 257.27$, $p < 0.00001$
TPD ♀	144	89	58	$\chi^2(2:1:1) = 3.301$, $p = 0.192$
TPD ♀	159	109	71	$\chi^2(2:1:1) = 5.234$, $p = 0.073$
TPD ♀	106	60	54	$\chi^2(2:1:1) = 0.193$, $p = 0.908$

Extended Data Table 2: Genotypic Segregation Ratios for Reciprocal *TPD* Crosses

Parent	***tpd1; tpd2***	***Tpd2+/-***	***Tpd/++***	***Tpd1+/-***	Chi-square
TPD ♂	0	0	142	0	$\chi^2(1:1:1:1) = 162.44$, $p < 0.00001$
TPD ♂	0	0	179	0	$\chi^2(1:1:1:1) = 204.41$, $p < 0.00001$
TPD ♂	0	0	219	0	$\chi^2(1:1:1:1) = 254.37$, $p < 0.00001$
TPD ♀	81	63	89	58	$\chi^2(1:1:1:1) = 4.447$, $p = 0.217$
TPD ♀	69	90	109	71	$\chi^2(1:1:1:1) = 6.029$, $p = 0.110$
TPD ♀	51	55	60	54	$\chi^2(1:1:1:1) = 0.378$, $p = 0.945$

Extended Data Table 3: Genome Assembly Metrics. Values calculated on scaffolded assemblies with contigs split by 10 or more consecutive Ns. Repeat content determined by RepeatMasker with the combined MTEC repeat library “maizeTE02052020” distributed at https://github.com/oushujun/MTEC.

	**TPD**<br>**TPD 1.0 2021**	**W22**<br>**W22 Reference NRGENE-2.0 2018**
**Contigs**		
#	945	49,111
Longest (Kbp)	44,353	831
N50 (Kbp)	7,744	88
**Scaffolds**		
# > 10Mbp	10	10
Span (Mbp)	2,112	2,134
Longest (Mbp)	306	311
Gap bases (%)	0.02	1.90
**Annotations**		
Coding Genes (W22 liftover)	40,564	40,961
Interspersed Repeats (%)	81.37	80.04
CentC masked (Kbp)	2,876	137

Extended Data Table 4: Empirical *p*-values for selection scan with a windowed weighted F_{ST} statistic for *Teosinte Pollen Drive*-linked (*TPD*-linked) regions and a validation set of domestication genes. See Extended Data Table 5 for gene coordinates. Empirical *p*-values for each gene are shown for the overlapping window with the lowest *p*-value. Significant *p*-values <0.05 are indicated with an asterisk for both unadjusted and Bonferroni-adjusted values accounting for each population pair tested.

Group	Gene	***mexicana* - Landrace**	***parviglumis* - Landrace**	**Landrace - Modern**	***mexicana* - Modern**	***parviglumis* - Modern**
		**Windowed F_{ST} empirical *p*-values**				
TPD-linked	*dcl2*	0.321	0.584	0.479	0.132	0.345
TPD-linked	hairpin	0.305	0.503	0.742	0.106	0.255
TPD-linked	*rdm1*	0.510	0.660	0.326	0.585	0.802
TPD-linked	*tdr1*	0.263	0.324	0.166	0.096	0.124
Domestication	*gt1*	0.128	0.058	0.020*	0.213	0.164
Domestication	*tb1*	0.016*	0.110	0.329	0.004*	0.047*
Domestication	*tga1*	0.032*	0.003*	0.356	0.021*	0.004*
Domestication	*zagl1*	0.025*	0.001*	0.201	0.003*	0.001*
		**Windowed F_{ST} empirical adjusted *p*-values**				
TPD-linked	*dcl2*	1.000	1.000	1.000	0.662	1.000
TPD-linked	hairpin	1.000	1.000	1.000	0.529	1.000
TPD-linked	*rdm1*	1.000	1.000	1.000	1.000	1.000
TPD-linked	*tdr1*	1.000	1.000	0.828	0.482	0.622
Domestication	*gt1*	0.638	0.290	0.100	1.000	0.822
Domestication	*tb1*	0.082	0.551	1.000	0.021*	0.236
Domestication	*tga1*	0.158	0.016*	1.000	0.105	0.020*
Domestication	*zagl1*	0.123	0.004*	1.000	0.013*	0.003*

Extended Data Table 5: Selection scan with a windowed |iHS| statistic in *Teosinte Pollen Drive*-linked (*TPD*-linked) regions and a validation set of domestication genes in teosinte and maize populations. Counts of individual significant (p<0.05) SNPs as well as empirical p-values for 10kb windows are shown. Genomic coordinates for *dcl2* (Zm00001eb219690), *tdr1* (Zm00001eb224090) and *rdm1* (Zm00001eb275620) are for the public maize B73 NAMv5 reference genome and annotation from MaizeGDB. Coordinates for the domestion genes are based on the same assembly and annotation. Significant p-values <0.05 are indicated with an asterisk. SNP counts and per window p-values are shown as unadjusted and Bonferroni-adjusted values accounting for each population tested. Cases in which there was insufficient data to calculate a p-value are shown as "NA".

			mexicana	***parviglumis***	**Landrace**	**Modern**
**Group**	**Gene**	**Genomic coordinates**	**Count of |iHS| significant SNPs**			
TPD-linked	*dcl2*	Chr5:20,831,070-20,848,118 (-)	46	6	1	18
TPD-linked	*tdr1*	Chr5:40,759,118-40,761,756 (-)	1	0	0	0
TPD-linked	hairpin	Chr5:95,990,889-96,002,706 (+)	4	30	1	0
TPD-linked	*rdm1*	Chr6:107,817,218-107,820,394 (+)	0	2	0	14
Domestication	*zagl1*	Chr5:20,831,070-20,848,118 (-)	48	40	0	0
Domestication	*gt1*	Chr5:40,759,118-40,761,756 (-)	0	0	0	3
Domestication	*tb1*	Chr5:95,990,889-96,002,706 (+)	5	2	2	0
Domestication	*tga1*	chr1:272330564-272332648 (+)	2	0	0	0
			**Count of |iHS| adjusted significant SNPs**			
TPD-linked	*dcl2*	Chr5:20,831,070-20,848,118 (-)	10	0	0	0
TPD-linked	*tdr1*	Chr5:40,759,118-40,761,756 (-)	0	0	0	0
TPD-linked	hairpin	Chr5:95,990,889-96,002,706 (+)	0	10	0	0
TPD-linked	*rdm1*	Chr6:107,817,218-107,820,394 (+)	0	0	0	6
Domestication	*zagl1*	Chr1:4932248-4948340 (-)	0	12	0	0
Domestication	*gt1*	chr1:23433554-23435122 (+)	0	0	0	3
Domestication	*tb1*	chr1:272330564-272332648 (+)	4	1	2	0
Domestication	*tga1*	chr4:46647932-46652896 (+)	0	0	0	0
			**Windowed |iHS| empirical *p*-values**			
			**Windowed |iHS| empirical *p*-values**			
TPD-linked	*dcl2*	Chr5:20,831,070-20,848,118 (-)	0.008*	0.341	0.316	0.079
TPD-linked	*tdr1*	Chr5:40,759,118-40,761,756 (-)	0.377	0.649	0.759	0.468
TPD-linked	hairpin	Chr5:95,990,889-96,002,706 (+)	0.216	0.171	0.493	0.215

TPD-linked	*rdm1*	Chr6:107,817,218-107,820,394 (+)	0.421	0.414	0.737	0.021*
Domestication	*zagl1*	Chr5:20,831,070-20,848,118 (-)	0.020*	0.039*	NA	NA
Domestication	*gt1*	Chr5:40,759,118-40,761,756 (-)	0.913	0.373	0.780	0.410
Domestication	*tb1*	Chr5:95,990,889-96,002,706 (+)	0.021*	0.116	0.054	0.188
Domestication	*tga1*	chr1:272330564-272332648 (+)	0.315	0.477	0.641	0.673
			**Windowed |iHS| empirical adjusted *p*-values**			
TPD-linked	*dcl2*	Chr5:20,831,070-20,848,118 (-)	0.031*	1.000	1.000	0.314
TPD-linked	*tdr1*	Chr5:40,759,118-40,761,756 (-)	1.000	1.000	1.000	1.000
TPD-linked	hairpin	Chr5:95,990,889-96,002,706 (+)	0.863	0.686	1.000	0.861
TPD-linked	*rdm1*	Chr6:107,817,218-107,820,394 (+)	1.000	1.000	1.000	0.085
Domestication	*zagl1*	Chr1:4932248-4948340 (-)	0.080	0.156	NA	NA
Domestication	*gt1*	chr1:23433554-23435122 (+)	1.000	1.000	1.000	1.000
Domestication	*tb1*	chr1:272330564-272332648 (+)	0.082	0.465	0.217	0.753
Domestication	*tga1*	chr4:46647932-46652896 (+)	1.000	1.000	1.000	1.000
