# Supplementary material for: Teosinte Pollen Drive guides maize domestication and evolution by RNAi
Source: bioRxiv. 2023 Aug 3:2023.07.12.548689. Originally published 2023 Jul 13. Preprint. [Version 2] doi: 10.1101/2023.07.12.548689 (PMC10370002; doi:10.1101/2023.07.12.548689)
Supplement: Supplementary file 2 [file media-2.pdf]

**Supplementary tables for**

# *Teosinte Pollen Drive* guides maize domestication and evolution by RNAi.

## Authors:

Benjamin Berube[1], Evan Ernst[1], Jonathan Cahn[1], Benjamin Roche[1], Cristiane de Santis Alves[1], Jason Lynn[1], Armin Scheben[2], Adam Siepel[2], Jeffrey Ross-Ibarra[3], Jerry Kermicle[4] and Rob Martienssen[1*]

[1] Howard Hughes Medical Institute, Cold Spring Harbor Laboratory, Cold Spring Harbor NY11724
[2] Simons Center for Quantitative Biology, Cold Spring Harbor Laboratory, Cold Spring Harbor NY11724
[3] Dept. of Evolution & Ecology, Center for Population Biology and Genome Center, University of California, Davis CA
[4] Laboratory of Genetics, University of Wisconsin, Madison WI

**Supplementary Table 1: Genotyping markers used in this study.** The "type" column refers to the general marker design associated with the primer sequence. RFLP markers will denote the associated restriction enzyme in the "type" column.

| Name | Type | Sequence (5'-3') | WT | Mut/*TPD* |
|---|---|---|---|---|
| *dcl2^T*f | BstNI | AGCGCCATTTACAATTTCAGCA | 183, 211 | 394 |
| *dcl2^T*-r | BstNI | TGTTGCCAGTGAATCAGCACTA | 183, 211 | 394 |
| m5.0-f | SSLP | TTAGTAGTGTCCTGGCGCTC | 295 | 165 |
| m5.0-r | SSLP | GTGAGGGACTAGGGCATGTG | 295 | 165 |
| m5.1-f | SSLP | CCTGCATAGAGATGCCATCAA | 310 | 180 |
| m5.1-r | SSLP | CGACGACGACTCATCCACGA | 310 | 180 |
| m5.2-f | SSLP | TGTCTTCCTCCAACTGTGCT | 312 | 211 |
| m5.2-r | SSLP | ACTGCCCAAAGAGCATGTGT | 312 | 211 |
| m5.3-f | MfeI | AATGGTGTTTTCCTTGGCATTCA | 253, 249 | 502 |
| m5.3-r | MfeI | GTACCATGCACTCATCCCGAA | 253, 249 | 502 |
| m5.4-f | TseI | GGATCATGGAGTGCGTGCAG | 450 | 161, 289 |
| m5.4-r | TseI | AACCAGCGCTCCTCAAAGTT | 450 | 161, 289 |
| m6.0-f | SSLP | ACTGAGTAACCAATGCCAGA | 445 | 363 |
| m6.0-r | SSLP | GCAGCCTTCCAGTTCCTGTA | 445 | 363 |
| m6.1-f | SSLP | GATCTACTTGCACGAGAGCACC | 495 | 374 |
| m6.1-r | SSLP | CGGAGTAATTTCCTTGGGACA | 495 | 374 |
| m6.2-f | AflIII | CTACCAACTGCTCCTGAGATGG | 116, 96 | 212 |
| m6.2-r | AflIII | CGTTGACGAATATTGATTGTAGCCA | 116, 96 | 212 |
| m6.3-f | NdeI | TTGCTCCAACCTTGTCACCT | 124, 100 | 224 |
| m6.3-r | NdeI | GCTATCCGCAAACAGCGAGA | 124, 100 | 224 |
| m6.4-f | BccI | TCCTTCTCCTCTTCCCTCGG | 250 | 110, 140 |
| m6.4-r | BccI | AGGAACCCTGTTTGACGATCT | 250 | 110, 140 |
| m6.5-f | BglIII | TCCACAGAAGGACAGCAAAAGGA | 174, 65 | 239 |
| m6.5-r | BglIII | TAGGGTTTGTTGTGGCTGCT | 174, 65 | 239 |
| m6.6-f | PvuII | GGCCAAGTTGTTCAAGAAGCAT | 165, 85 | 254 |
| m6.6-r | PvuII | GCGTGGCCCCTTCTCTTATT | 165, 85 | 254 |
| m6.7-f | NdeI | GGGCATCGTGTCATTGAAGG | 231 | 115, 116 |
| m6.7-r | NdeI | TGCAACCTCTCAGGTCTAAG | 231 | 115, 116 |
| *lbl-rgd1*-f | MwoI | GCCCATCTGGATCTGAAGTC | 262, 227 | 489 |
| *lbl-rgd1*-r | MwoI | TTGGTGGCCACACTATCTCA | 262, 227 | 489 |
| *dcl2mu1*-f | Mu insertion | GTGTCCGCGTTCCAGAAGTC | 906 | 800 |
| *dcl2mu1*-r | Mu insertion | TAAAGGTTGTCCATTGGGCGTT | 906 | 800 |
| TIR4 | TIR | GCCTCCATTTCGTCGAATCCC | - | - |
| TIR6 | TIR | GCCTCTATTTCGTCGAATCCG | - | - |
| Gdsl-f | HiII check | GAGCGTCTCCTTCAACCCAA | 983 | - |
| Gdsl-r | HiII check | TCCTACTCCTCAGTTGGGGG | 983 | - |
| Dcl2-f | HiII check | GGCCTAGAATTCAGTTGCGG | 717 | - |
| Dcl2-r | HiII check | GAACACGCTTGTGTTCCTCG | 717 | - |

**Supplementary Table 2: RT-qPCR primers used in this study.** All RT-qPCR primers used in this study were designed to bridge exon junctions (if present) in order to increase specificity. Expected amplicon sizes corresponding to a cDNA or gDNA template are listed.

| Name | Target | Sequence | cDNA | gDNA |
|---|---|---|---|---|
| *Dcl2q-1f* | *Dcl2* – exon 1,2 | CCCAAAAGGACACACAGCTTTC | 79 | 159 |
| *Dcl2q-1r* | *Dcl2* – exon 1,2 | GCATATCTGATCTTGGAGTATGGC | 79 | 159 |
| *Dcl2q-2f* | *Dcl2* – exon 5,6 | TCGACAAAAACAATGCATCTCAAAT | 104 | 658 |
| *Dcl2q-2r* | *Dcl2* – exon 5,6 | GCTGAGGATCTGCAAGATGG | 104 | 658 |
| *Dcl2q-3f* | *Dcl2* – exon 18,19 | CATTCTGAAGGGTGTCGGGT | 174 | 417 |
| *Dcl2q-3r* | *Dcl2* – exon 18,19 | GGTCCATCACATGGTTGGGAA | 174 | 417 |
| *Gdslq-1f* | *Gdsl* – exon 1,2 | GAGCGTCTCCTTCAACCCAA | 81 | 249 |
| *Gdslq-1r* | *Gdsl* – exon 1,2 | ACAAGGCTACTGGCAGGTTC | 81 | 249 |
| *Gdslq-2f* | *Gdsl* – exon 2,3 | CCCACCTTCGCCTTGTACTC | 153 | 250 |
| *Gdslq-2r* | *Gdsl* – exon 2,3 | CCTGAACAAGAGCCTCACCC | 153 | 250 |
| *Elfa9q-1f* | *Efla9* – exon 1,2 | CAAGCTGACTGTGCTGTTCTTA | 181 | 928 |
| *Elfa9q-1r* | *Efla9* – exon 1,2 | GCCTTTGAATACTTGGGTGTAGTAG | 181 | 928 |
| *Elfa9q-2f* | *Efla9* – exon 1,2 | CCAAGCTGACTGTGCTGTTCTT | 198 | 945 |
| *Elfa9q-2r* | *Efla9* – exon 1,2 | AATCTCATCATAACGGGCCTTTGAA | 198 | 945 |
| *Rdr6q-1f* | *Rdr6* – no exons | TGTTTTGCTTCAGCTGGGGA | 128 | 128 |
| *Rdr6q-1r* | *Rdr6* – no exons | CACGCGAATGAAGCATCTGG | 128 | 128 |
| *Rdr6q-2f* | *Rdr6* – no exons | GCCTCTCACCTTTCTTGGGG | 74 | 74 |
| *Rdr6q-2r* | *Rdr6* – no exons | CCTTGAAGCTGTTGATGTGCC | 74 | 74 |
| *Rgd1q-1f* | *Rgd1* – exon 5,6 | GCCACATCCTTGACTTGGCA | 119 | 319 |
| *Rgd1q-1r* | *Rgd1* – exon 5,6 | TGCAGGAAGAGCGCTCAAAG | 119 | 319 |
| *Rgd1q-2f* | *Rgd1* – exon 4,6 | CGCTCTTCCTGCAACAACTT | 130 | 378 |
| *Rgd1q-2r* | *Rgd1* – exon 4,6 | GAGAAGCACATGGAGTACGAGG | 130 | 378 |
| *Ago1e-1f* | *Ago1e* – exon 19,20 | TTCTACCTGTGCAGCCATGC | 103 | 172 |
| *Ago1e-1r* | *Ago1e* – exon 19,20 | GAGTTTGCAACCCATCAGCC | 103 | 172 |
| *Ago1e-1f* | *Ago1e* – exon 10,11 | GCCAAAAATTGGCCAGTGGA | 128 | 515 |
| *Ago1e-1r* | *Ago1e* – exon 10,11 | TCATGACAGAAACTCCCGGC | 128 | 515 |
